# NeoRdRp: A Comprehensive Dataset for Identifying RNA-dependent RNA Polymerases of Various RNA Viruses from Metatranscriptomic Data

**DOI:** 10.1264/jsme2.ME22001

**Published:** 2022-08-24

**Authors:** Shoichi Sakaguchi, Syun-ichi Urayama, Yoshihiro Takaki, Kensuke Hirosuna, Hong Wu, Youichi Suzuki, Takuro Nunoura, Takashi Nakano, So Nakagawa

**Affiliations:** 1 Department of Microbiology and Infection Control, Faculty of Medicine, Osaka Medical and Pharmaceutical University; 2 Laboratory of Fungal Interaction and Molecular Biology (donated by IFO), Department of Life and Environmental Sciences, University of Tsukuba; 3 Super-cuttingedge Grand and Advanced Research (SUGAR) Program, Japan Agency for Marine-Earth Science and Technology (JAMSTEC); 4 Medical School, Osaka Medical and Pharmaceutical University, Takatsuki, Japan; 5 Research Center for Bioscience and Nanoscience (CeBN), Japan Agency for Marine-Earth Science and Technology (JAMSTEC); 6 Department of Molecular Life Science, Tokai University School of Medicine

**Keywords:** RNA virome, RNA-dependent RNA polymerase, hidden Markov model, metatranscriptome

## Abstract

RNA viruses are distributed throughout various environments, and most have recently been identified by metatranscriptome sequencing. However, due to the high nucleotide diversity of RNA viruses, it is still challenging to identify novel RNA viruses from metatranscriptome data. To overcome this issue, we created a dataset of RNA-dependent RNA polymerase (RdRp) domains that are essential for all RNA viruses belonging to *Orthornavirae*. Genes with RdRp domains from various RNA viruses were clustered based on amino acid sequence similarities. A multiple sequence alignment was generated for each cluster, and a hidden Markov model (HMM) profile was created when the number of sequences was greater than three. We further refined 426 HMM profiles by detecting RefSeq RNA virus sequences and subsequently combined the hit sequences with the RdRp domains. As a result, 1,182 HMM profiles were generated from 12,502 RdRp domain sequences, and the dataset was named NeoRdRp. The majority of NeoRdRp HMM profiles successfully detected RdRp domains, specifically in the UniProt dataset. Furthermore, we compared the NeoRdRp dataset with two previously reported methods for RNA virus detection using metatranscriptome sequencing data. Our methods successfully identified the majority of RNA viruses in the datasets; however, some RNA viruses were not detected, similar to the other two methods. NeoRdRp may be repeatedly improved by the addition of new RdRp sequences and is applicable as a system for detecting various RNA viruses from diverse metatranscriptome data.

Many RNA viruses have been identified by the deep sequencing of RNAs from diverse environmental samples (*i.e.*, metatranscriptome) (Shi *et al.*, 2018; Sakaguchi *et al.*, 2020; Orba *et al.*, 2021). Various metatranscriptomic ana­lyses have revealed that RNA viruses, similar to human infectious viruses, such as coronaviruses, orthomyxoviruses, and filoviruses, are present even in non-mammalian species, including fishes (Shi *et al.*, 2018). However, identifying RNA virus-derived sequences from metatranscriptome data is challenging because of the low abundance of viral RNA in sequencing reads and high nucleotide diversity among RNA viruses (Cobbin *et al.*, 2021). Therefore, we developed a method of double-stranded RNA (dsRNA) sequencing named “fragmented and primer-ligated dsRNA sequencing” (FLDS) to enrich RNA virus sequences in the cellular transcriptome (Urayama *et al.*, 2016, 2018; Hirai *et al.*, 2021). Long dsRNAs are rare in eukaryotic cells; therefore, they are mainly derived from the genomes of dsRNA viruses or replicative intermediates of single-strand RNA (ssRNA) viruses (Kumar and Carmichael, 1998). Moreover, FLDS enables us to obtain entire dsRNA sequences, including both termi, and to reconstruct the multi-segmented genomes of RNA viruses based on terminal sequence similarities (Urayama *et al.*, 2016). Accordingly, FLDS may enrich potential RNA virus genomes even though their sequences are dissimilar to known RNA viruses. However, it remains difficult to reach conclusions regarding RNA viral genomes based on FLDS data if genes specific to RNA viruses are not found.

Viruses that utilize RNA as genetic material form a monophyletic group named *Riboviria* based on the classification system of ICTV (International Committee on Taxonomy of Viruses, https://talk.ictvonline.org/). *Orthornavirae* is a major kingdom of *Riboviria* containing all dsRNA and ssRNA viruses, but excluding retroviruses, which harbor RNA-dependent RNA polymerase (RdRp). RdRp genes may be used as markers for most RNA viruses; however, their nucleotide and amino acid sequences markedly vary, and some have been identified as fusion genes with other protein motifs (Černý *et al.*, 2014; Wolf *et al.*, 2018; Koonin *et al.*, 2020, 2021). It is often challenging to identify RNA virus genes, including the RdRp gene, even in cases in which potentially complete viral genome sequences are obtained by FLDS based on a similarity search using BLAST (Basic Local Alignment Search Tool) with major amino acid databases (*e.g.*, nr database or RefSeq database) (Jia and Gong, 2019; Urayama *et al.*, 2020).

There are eight conserved amino acid motifs in RdRp domain sequences; the sixth motif containing three amino acid residues, GDD, is highly conserved (Koonin, 1991). Based on these RdRp sequence motifs, Wolf and his colleagues inferred the phylogeny of 4,617 RNA viruses (Wolf *et al.*, 2018). However, the accuracy of the multiple alignment of all RdRp amino acid sequences has been disputed due to their diversity (Holmes and Duchêne, 2019; Wolf *et al.*, 2019). Therefore, to obtain multiple alignments of RdRp sequences that are reliable, it is necessary to create multiple sequence alignments for each group defined by a shared high sequence similarity.

The present study collected RdRp domain sequences from 23,410 RNA viruses and clustered them based on their similarity to build 1,182 hidden Markov model (HMM) profiles. Since a HMM profile is based on a multiple sequence alignment, it may identify distantly related sequences more efficiently than a pairwise-based search (Mistry *et al.*, 2021). Several bioinformatics approaches using HMM have been devised to identify more distantly related viruses, such as VirSorter2 (Guo *et al.*, 2021) and RVDB-prot (Goodacre *et al.*, 2018; Bigot *et al.*, 2019). Since these datasets were based on various proteins in RNA and DNA viruses (Goodacre *et al.*, 2018; Bigot *et al.*, 2019), the protein motifs identified by these programs are not necessarily specific to RNA viruses. Furthermore, this study analyzed the RdRp sequences of marine RNA viruses obtained by FLDS and revealed that our method successfully detected RdRp domains more efficiently than the two approaches described above (*i.e.*, VirSorter2 and RVDB-prot). The RdRp construction bioinformatics pipeline and constructed RdRp dataset, including annotation, are publicly available online (https://github.com/shoichisakaguchi/NeoRdRp).

## Materials and Methods

### Datasets

A total of 4,620 amino acid sequences containing RdRp domains annotated by Wolf and his colleagues (Wolf *et al.*, 2018) were downloaded from the National Center for Biotechnology Information (NCBI) GenBank database. Additionally, we obtained 18,790 amino acid sequences of 4,239 RNA viruses from the NCBI Virus database (https://www.ncbi.nlm.nih.gov/labs/virus/vssi/) on June 9, 2021. These datasets are referred to as Wolf-RdRps and NCBI-RNA-virus, respectively, in the present study.

The UniProt Knowledgebase (UniProtKB) containing manually curated protein sequences and functional information concerning 565,928 genes (UniProtKB Reviewed [Swiss-Prot] 2021-05-11) (Boutet *et al.*, 2016) was used to validate and annotate our dataset. The gene ontology (GO) molecular function of RdRp genes was GO:0003968 (RNA-directed 5′-3′ RNA polymerase activity). In the UniProtKB database, 1,027 out of 565,928 amino acid sequences were categorized as GO:0003968; 836 of these were validated as RdRp-containing genes (Supplementary Table S1).

In addition, potential RNA virus genome sequences obtained from the marine metatranscriptomic ana­lysis by fragmented and primer ligated dsRNA sequencing (FLDS) were used as a benchmark dataset (Urayama *et al.*, 2018). This dataset contains 228 RdRp domains in 1,143 potentially complete RNA virus genomes. Open reading frames (ORFs) longer than 30 nucleotides (starting with any sense codon) were predicted for each genome using ORFfinder version 0.4.3, with the following options: -ml 30 -s‍ ‍2 (available at https://www.ncbi.nlm.nih.gov/orffinder/).

### Clustering based on amino acid sequence similarities

Amino acid sequences were clustered using CD-HIT program version 4.7 (Li and Godzik, 2006; Fu *et al.*, 2012) with the following criteria: a similarity threshold of 60% and word size of 4. A multiple sequence alignment was generated using the L-INS-i program in MAFFT version 7.450 (Katoh and Standley, 2013). Regions with solid statistical evidence of shared homology for each multiple sequence alignment were selected by replacing an amino acid in the uncertainty regions with a gap (-) using Divvier version 1.0, with the default parameters (Ali *et al.*, 2019). In the multiple sequence alignment, if five or more amino acid positions followed by more than 25% of the sequences consisted of gaps, the region was defined as a boundary between two different domains. The multiple sequence alignment was then split at the boundary using our in-house script. Multiple alignments of amino acid sequences were visualized using WebLogo version 3.7.8 with the following options: -c chemistry -U probability (Crooks *et al.*, 2004).

### Creation of Hidden Markov Model (HMM) profiles

A HMM profile was built for each multiple sequence alignment using hmmbuild, part of the HMMER3 package version 3.1b2 (Eddy, 2011) with default parameters. All HMM profiles were then compressed into a HMMER3 searchable database using hmmpress in the HMMER3 package with default parameters.

### HMM and BLAST search

All HMM searches with HMM profiles were performed using the hmmsearch program from the HMMER3 package with default parameters and three sequence threshold E-values: “-E 1E-10”, “-E 1E-20”, and “-E 1E-30”. Two hits in a sequence with gap lengths less than 500 amino acids were merged using BEDTools version 2.30.0 (Quinlan and Hall, 2010) with the “merge -d 500” option (Supplementary Fig. S1). The BLASTp program from the BLAST+ software suite version 2.12.0 (Camacho *et al.*, 2009) was used with the “-evalue 1e-10”, “-evalue 1e-20”, and “-evalue 1e-30” options.

### VirSorter2 and RVDB-prot

VirSorter2 (Guo *et al.*, 2021) was used with the option “--‍include-groups RNA,” targeting the search group set to RNA viruses only. The HMM profiles obtained from Reference Viral Protein DataBase (RVDB-prot) version 22.0 (Bigot *et al.*, 2019) were used with the hmmsearch program.

### Computer resource

A workstation PC (CPU, Intel Core i9-7980XE 2.60 GHz; Memory, 128 GB; Storage 19 TB SSD) with Linux OS (Ubuntu 18.04.6 LTS) was used to benchmark each bioinformatics program.

## Results

### Constructing RdRp HMM profiles

**Fig. 1.**
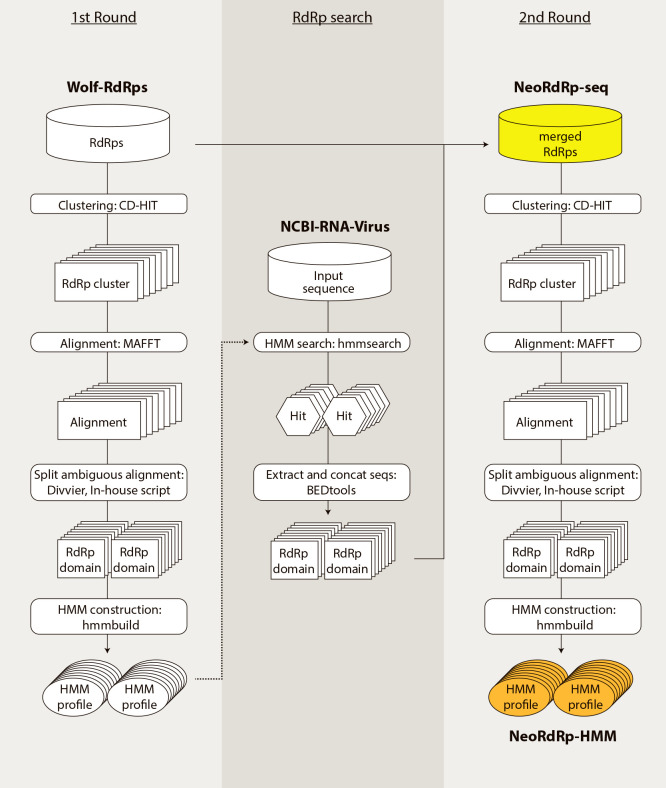
A schematic workflow of NeoRdRp-HMM construction (1st Round, left) Amino acid sequences containing RdRp domains in Wolf-RdRps data were clustered based on similarities. A multiple sequence alignment was constructed for each cluster and processed to create HMM profiles. (RdRp search, middle) HMM profiles were used to detect the RdRp domain of amino acid sequences in NCBI-RNA-Virus data. (2nd Round, right) The detected RdRp domain sequences were merged with Wolf-RdRps data, which were clustered and processed to create HMM profiles for the detection of RdRp. The dataset names used in this study are in bold. Yellow, NeoRdRp-seq; orange, NeoRdRp-HMM.

The schematic procedure for constructing RdRp HMM profiles is shown in Fig. 1. The details of datasets and software used for the ana­lysis are described in the Materials and Methods. We initially obtained the amino acid sequences of 4,620 RdRp genes and those with the RdRp domain selected by Wolf and his colleagues (named Wolf-RdRps) (Wolf *et al.*, 2018). We then clustered 4,620 amino acid sequences based on their sequence similarities and obtained 3,190 clusters. Clusters containing more than three sequences (262 clusters in total) were used to construct HMM profiles. The amino acid sequences for each cluster were aligned. Since genes including the RdRp domain may contain multiple other domains, unreliable regions in multiple sequence alignments were replaced with gaps. In the multiple sequence alignment, if five or more amino acid positions followed by more than 25% of the sequences consisted of gaps, the region was split there and divided into two multiple alignments. A multiple sequence alignment consisting of more than nine amino acids was subsequently defined as a domain. A HMM profile was created for each domain, and 426 HMM profiles were obtained. The script for the above-described bioinformatics procedure is available at the following website: https://github.com/shoichisakaguchi/NeoRdRp.

**Fig. 2.**
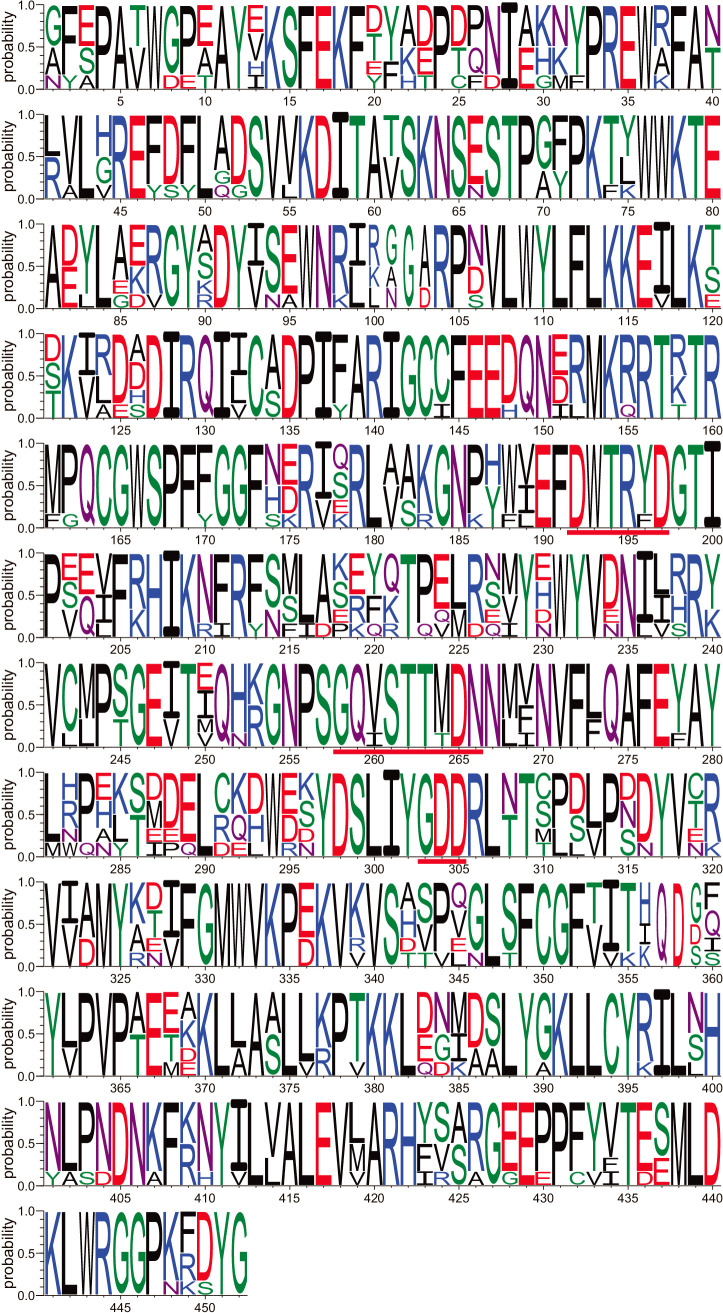
An example of a multiple sequence alignment of amino acid sequences including the RdRp domain. The multiple sequence alignment of the HMM profile of 1981.alnta.divvy_RDRP_0.25_923-1375 (including seven sequences) was visualized using Weblogo software. The height represents the probability at each site, and the width is proportional to the number of sequences. Underlined areas in red indicate the well-conserved DxxxxD, GxxxTxxxN, and GDD motifs in the RdRp domain at positions 192–197, 258–266, and 303–305, respectively.

We also conducted comprehensive similarity searches using 426 HMM profiles with 18,790 amino acid sequences of 4,239 RefSeq RNA virus genomes downloaded from NCBI Virus (named NCBI-RNA-Virus). A total of 7,463 viral amino acid sequences were obtained through the HMM search. The amino acid sequences obtained were combined with Wolf-RdRps, resulting in 12,502 amino acid sequences named “NeoRdRp-seq”. By applying the same procedure to construct HMM (1st round shown in Fig. 1), we clustered the NeoRdRp-seq dataset based on amino acid sequence similarities, resulting in 1,092 clusters (including 8,516 sequences). Note that 2,753 clusters (including 3,986 sequences) containing fewer than three sequences for each were excluded from the HMM profile construction. The 1,092 clusters were processed using the same procedures, and 1,182 domains were extracted. Fig. 2 shows an example of the domain clearly indicating the well-conserved protein motifs, DxxxxD, GxxxTxxxN, and GDD (Koonin, 1991). A HMM profile was created for each domain, and 1,182 HMM profiles were obtained, which were named “NeoRdRp-HMM” and are available at https://github.com/shoichisakaguchi/NeoRdRp.

### Evaluating NeoRdRp-HMM and NeoRdRp-seq

The amino acid sequences and annotation information obtained from the UniProtKB Reviewed database were used to evaluate NeoRdRp-HMM and NeoRdRp-seq. The dataset consisted of 565,928 amino acid sequences, including 836 genes with RdRp domains (see Materials and Methods and Supplementary Table S1). With 565,928 amino acid sequences as queries, we conducted comprehensive searches using hmmsearch or BLASTp, targeting NeoRdRp-HMM or NeoRdRp-seq, respectively, with three different threshold E-values: 1E-10, 1E-20, and 1E-30. The resulting data are summarized in Supplementary Table S2, and the recall and precision rates calculated from them are summarized in Table 1. In both methods and datasets, smaller E-values were associated with fewer true-positive and false-positive hits. NeoRdRp-seq generally identified more genes with the RdRp domain with more false-positive sequences than NeoRdRp-HMM. For example, with an E-value 1E-10 threshold, 813 and 824 out of 836 genes with the RdRp domain and 246 and 1,316 out of 564,418 non-RdRp genes were detected using the NeoRdRp-HMM and NeoRdRp-seq datasets, respectively. Furthermore, our dataset identified genes with the RdRp domain from various RNA viruses, but not 12 genes with the RdRp domain (1.4%) derived from nine Birnaviridae, two Reoviridae, and one Paramyxoviridae.

We also annotated NeoRdRp-HMM profiles based on the UniProtKB annotation of Gene Ontology (GO; mole­cular function) with an E-value 1E-10 threshold. Out of 1,182 HMM profiles, 1,012 were evaluated; each hit was scored 1 if “RNA-directed 5′-3′ RNA polymerase activity” (GO:0003968) was included and 0 if it was excluded. We then calculated the mean value of the scores for each NeoRdRp-HMM profile, as summarized in Supplementary Table S3. The mean score of the profiles was 0.92, suggesting that most NeoRdRp-HMM profiles annotated using the UniProtKB search were derived from the RdRp domain sequences of RNA viruses. However, we also found that 13 HMM profiles (1.3%) had a score of 0; RNA binding [GO:0003723], ATP binding [GO:0005524], helicase activity [GO:0004386], mRNA methyltransferase activity [GO:0008174], and hydrolase activity [GO:0016787] were shared among the seven HMM profiles. The results obtained indicated that these HMM profiles with a score of 0 detected proteins that interact with RNA, but do not contain RdRp domains.

### Comparison of RdRp identification

We benchmarked the ability of NeoRdRp-HMM and NeoRdRp-seq to identify RNA viruses using potentially complete RNA virus genome and genome segment sequences obtained by FLDS associated with marine RNA viromes (Urayama *et al.*, 2018). The data obtained comprised 1,143 nucleotide sequences. Among these, 112,108 open reading frames (ORFs) longer than 30 nucleotides (*i.e.*, 10 amino acids) were obtained (see Materials and Methods), including 228 sequences annotated as genes with the RdRp domain. The dataset of translated amino acid sequences was named FLDS-data.

Using FLDS-data, we evaluated NeoRdRp-HMM and NeoRdRp-seq using hmmsearch and BLASTp, respectively. NeoRdRp-HMM and NeoRdRp-seq were compared with E-values of 1E-10, 1E-20, and 1E-30; 181, 138, and 123 genes encoding the RdRp domain were found by both methods, respectively (Supplementary Fig. S2). Furthermore, with the 1E-10, 1E-20, and 1E-30 E-values, none, 5, and 2 RdRp domain sequences were detected by NeoRdRp-HMM only, whereas 18, 27, and 21 RdRp domain sequences were detected by NeoRdRp-seq, respectively. The difference in RdRp identification between the two methods may be explained by the advantage of the HMM search in the detection of diversified RdRp domain sequences and the lower coverage of the current HMM models because of the requirement of at least three sequences to create a new reliable HMM profile.

**Fig. 3.**
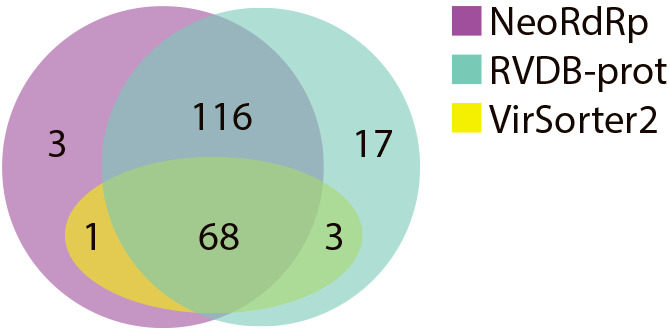
Venn diagram of three different methods for identifying RNA viruses. Annotated marine metatranscriptome data were searched using NeoRdRp (NeoRdRp-HMM and NeoRdRp-seq), RVDB-prot, and VirSorter2. The number in each region indicates the number of hits by the method(s); for example, three were found only in NeoRdRp. Purple, NeoRdRp (NeoRdRp-HMM and NeoRdRp-seq); green, RVDB-prot; yellow, VirSorter2.

We then merged the results of the NeoRdRp-HMM and NeoRdRp-seq searches into NeoRdRp. Since NeoRdRp-seq may detect large false-positive hits with a large E-value (Table 1), the threshold E-value of the BLASTp search with NeoRdRp-seq was fixed at 1E-30 in this search. VirSorter2 (Guo *et al.*, 2021) and hmmsearch with RVDB-prot (Goodacre *et al.*, 2018) were used for comparison. Note that since VirSorter2 only accepts nucleotide sequences as an input file, the original 1,143 nucleotide sequences of FLDS-data were used for VirSorter2. With a threshold E-value 1E-10 of hmmsearch, we analyzed the 228 genes with the RdRp domain using the three methods and detected them as follows: 68 by all three methods, 116 by NeoRdRp and RVDB-prot, one by NeoRdRp and VirSorter2, three by RdRp-prot and VirSorter2, three only by NeoRdRp, 17 only by RVDB-prot, and 20 were not detected by any methods (Fig. 3). Although all hits by NeoRdRp-HMM were also identified by RVDB-prot (Supplementary Fig. S3), viral genomes detected only by NeoRdRp were classified into the families *Flaviviridae* and *Reoviridae* and unclassified ssRNA positive-strand viruses based on HMM patterns and genomic structures. However, the viral genomes not detected by the three methods were recognized as members of *Reoviridae *and *Megabirnaviridae* (Urayama *et al.*, 2018).

We also found 13 possible RdRp-containing sequences in FLDS-data that were not previously annotated. According to the NeoRdRp-HMM annotation, 13 RdRp domain sequences were similar to those of *Picovirnaviridae*, unclassified viruses, or unclassified dsRNA viruses (Supplementary Table S4). These results suggest that our dataset is useful for detecting RdRps in metatranscriptome data that have been overlooked with previously published bioinformatic pipelines.

We also compared the computational time required to search for RNA viruses in FLDS-data using the three methods (Table 2): hmmsearch using NeoRdRp-HMM and BLASTp using NeoRdRp-seq took 1‍ ‍min 2‍ ‍s and 2‍ ‍min 56‍ ‍s, respectively (*i.e.*, 3‍ ‍min and 58‍ ‍s in total for the NeoRdRp search); hmmsearch using RVDB-prot took 7‍ ‍min 11 s; and the search using VirSorter took 11‍ ‍min 45 s. Furthermore, the database size required for each ana­lysis was 0.37 GB for NeoRdRp, 1.61 GB for RVDB-prot, and 11.3 GB for VirSorter2 (Table 2). These results indicated that the NeoRdRp dataset required a smaller computational capacity than the other methods tested.

## Discussion

Nucleic acid contamination from DNA viruses, hosts, and environments may occur when analyzing RNA viromes. To overcome this issue, techniques to selectively extract and sequence RNA virus-derived nucleic acids, such as the FLDS method (Urayama *et al.*, 2016; Hirai *et al.*, 2021), have been developed. However, it is still challenging to eliminate all contamination from sequencing data. Therefore, in the present study, RdRp genes, which are the hallmark of RNA viruses, were comprehensively obtained (NeoRdRp-seq), and the HMM profiles of the RdRp domains were constructed based on amino acid similarities (NeoRdRp-HMM) (Fig. 1). Using this dataset, we successfully obtained various genes encoding the RdRp domain from FLDS-data (Fig. 3), while those of some RNA viruses were not detected (Table 1 and Fig. 3). In addition, although NeoRdRp-seq may complement RdRp-HMM, a BLASTp search using NeoRdRp-seq generated a relatively large number of false-positive hits (Table 1). This is because an RdRp domain is sometimes encoded in a fusion gene, and NeoRdRp-seq includes the non-RdRp domain sequences of these fusion genes. To overcome this issue, repeating updates with additional RdRp sequences are necessary. In principle, as the number of RdRp sequences increases, the number of clusters used for HMM construction also increases, and the detection power must subsequently increase. We designed the pipeline used for NeoRdRp-HMM to create enhanced HMM profiles by incorporating newly detected RdRp sequences. The pipeline for HMM construction is available on GitHub (https://github.com/shoichisakaguchi/NeoRdRp). Therefore, users may modify NeoRdRp-HMM using their own sequences. Accordingly, future updates to NeoRdRp-HMM with additional identified RdRp sequences will reduce undetected RdRp sequences and false-positive hits.

The NeoRdRp dataset was based only on genes containing RdRp domains. Our approach has advantages in the accuracy of RNA virus identification from highly diversified metatranscriptome data, particularly for the presence of known viruses. Since NeoRdRp cannot identify non-RdRp genes in RNA viruses, the NeoRdRp dataset may be applied for RNA virus detection and annotation from metatranscriptome data as the following research design; the assembly of metatranscriptomic data, a subsequent ORF search for each contig, and the RdRp domain identification of each ORF using NeoRdRp-HMM. If more RNA virus sequences are required, further searches using NeoRdRp-seq are expected, but may produce more false-positive hits (Table 1 and Supplementary Table S2) and this process is computationally intensive (Table 2). Based on the combination of these methods and their scores, we may identify contigs containing the RdRp domain, suggesting that they are derived from RNA viruses. Furthermore, FLDS may identify multi-segmented genomes of RNA viruses based on terminal sequence similarities (Urayama *et al.*, 2016). Therefore, if one contig is found to contain an RdRp domain, other contigs with a similar sequence on either terminus are strongly implied to be derived from RNA viruses. Furthermore, in these contigs, other viral genes may be detected using different computational programs and datasets, including VirSorter2 and RVDB-prot.

In conclusion, NeoRdRp may detect various RdRps with a high degree of accuracy (Table 1 and Fig. 3), and the dataset is more compact than those obtained using other methods for RNA virome searches (Table 2). All sequence data, annotations, and bioinformatics pipelines are available on GitHub (https://github.com/shoichisakaguchi/NeoRdRp). Furthermore, by updating NeoRdRp with new RdRp sequences, it will be possible to find more RdRp sequences with higher accuracy levels from various metatranscriptome data, which may become an essential technique for discovering unknown RNA viruses.

## Citation

Sakaguchi, S., Urayama, S., Takaki, Y., Hirosuna, K., Wu, H., Suzuki, Y., et al. (2022) NeoRdRp: A Comprehensive Dataset for Identifying RNA-dependent RNA Polymerases of Various RNA Viruses from Metatranscriptomic Data. *Microbes Environ ***37**: ME22001.

https://doi.org/10.1264/jsme2.ME22001

## Supplementary Material

Supplementary Material 1

Supplementary Material 2

## Figures and Tables

**Table 1.** Recall and precision rates of NeoRdRp-HMM or NeoRdRp-seq searches for each E-value threshold.

	E-value ≤1E-10			E-value ≤1E-20			E-value ≤1E-30	
	Recall (%)	Precision (%)		Recall (%)	Precision (%)		Recall (%)	Precision (%)
NeoRdRp-HMM	97.2	76.8		96.8	84.8		96.2	85.0
NeoRdRp-seq	98.6	38.5		98.2	47.7		98.1	48.4
NeoRdRp	98.6	37.9		98.2	47.7		98.1	48.4

**Table 2.** Database sizes and processing times of three methods to search for RNA viruses in FLDS data.

Data	Tool	Database Type	Database Size (GB)	Speed
NeoRdRp-HMM/ NeoRdRp-Seq	hmmsearch/ BLASTp	HMM/ blast	0.36/ 0.02	1‍ ‍min 2 s/ 2‍ ‍min 56 s
RVDB-prot	hmmsearch	HMM	1.61	7‍ ‍min 11 s
VirSorter2	VirSorter2	VS2	11.13	11‍ ‍min 45 s
