# Supplementary material for: NeoRdRp: A Comprehensive Dataset for Identifying RNA-dependent RNA Polymerases of Various RNA Viruses from Metatranscriptomic Data
Source: Microbes Environ. 2022 Aug 24;37(3):ME22001. doi: 10.1264/jsme2.ME22001 (PMC9530720; doi:10.1264/jsme2.ME22001)

Supplementary Material

Supplementary Figure 1. Defining an RdRp domain.

When the detected sequence distance was less than 500 amino acid length in the hmmsearch results, we regarded them as a single region as a domain.

Supplementary Figure 2. Comparison of NeoRdRp-HMM and NeoRdRp-seq.

NeoRdRp-HMM and NeoRdRp-seq with an E-values of (a) 1E-10, (b) 1E-20, and (c) 1E-30 were compared. Pink, NeoRdRp-HMM; purple, NeoRdRp-seq.

Supplementary Figure 3. Comparison of NeoRdRp-HMM and RVDB-prot.

NeoRdRp-HMM and NeoRdRp-seq with E-values of (a) 1E-10, (b) 1E-20 and (c) 1E-30 were compared. Pink, NeoRdRp-HMM; purple, NeoRdRp-seq; green, RVDB-prot.

Supplementary Table 1. 836 RdRp genes in the UniProt KB database.

Supplementary Table 2. NeoRdRp-HMM or NeoRdRp-seq searches for each E-value threshold.

Supplementary Table 3. Summary of the scores for each NeoRdRp-HMM profile.

Supplementary Table 4. Unannotated RdRps in metatranscriptome data detected by NeoRdRp.

Supplementary Figure 1. Defining an RdRp domain

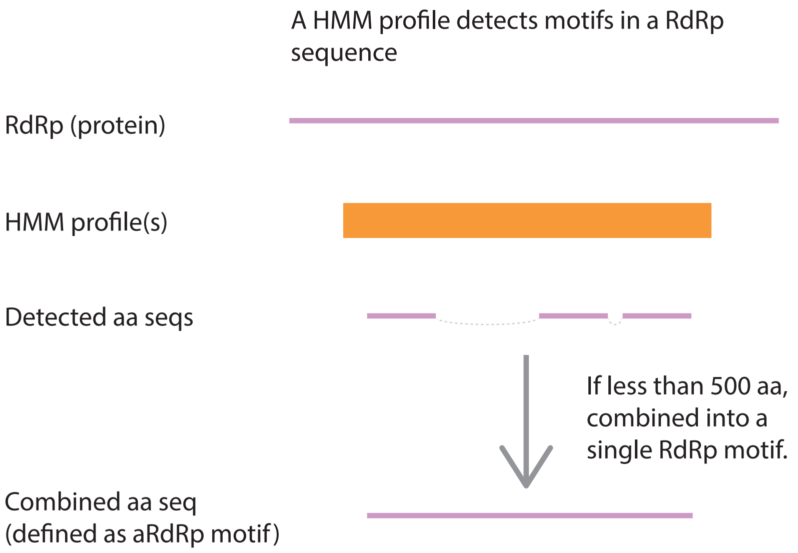

Supplementary Figure 2. Comparison of NeoRdRp-HMM and NeoRdRp-seq

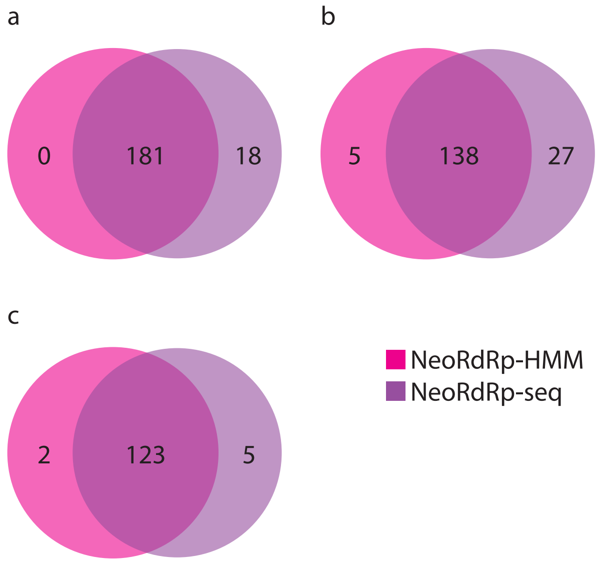

Supplementary Figure 3. Comparison of NeoRdRp-HMM and RVDB-prot

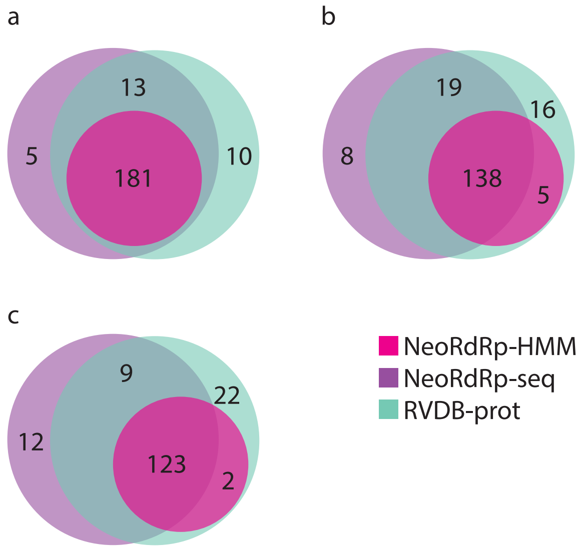

Supplement: Supplementary file 1 — Supplementary Material 1 [file 37_22001_s1.pdf]
